# A Comparative Analysis between Flexible Ureteroscopic Lithotripsy and Tubeless Percutaneous Nephrolithotomy in the Treatment of >15 mm Non-Obstructing Proximal Ureteral Stones

**DOI:** 10.3390/jcm12247541

**Published:** 2023-12-07

**Authors:** Yong Sun Choi, Samuel Ryan Sorkhi, Hyuk Jin Cho, Kang Sup Kim

**Affiliations:** 1 Department of Urology, Eunpyeong St. Mary’s Hospital, College of Medicine, The Catholic University of Korea, Seoul 06591, Republic of Korea; yschoi1008@catholic.ac.kr; 2 VA Medical Center San Diego, VA San Diego Healthcare System, San Diego, CA 92161, USA; samuelsorkhi@creighton.edu; 3 Department of Urology, Seoul St. Mary’s Hospital, College of Medicine, The Catholic University of Korea, Seoul 06591, Republic of Korea; a0969@catholic.ac.kr; 4 Department of Urology, Uijeongbu St. Mary’s Hospital, College of Medicine, The Catholic University of Korea, Uijeongbu 11765, Republic of Korea

**Keywords:** flexible ureteroscopic lithotripsy, tubeless percutaneous nephrolithotomy, proximal ureteral stones

## Abstract

Background: The proper surgical modality for large non-obstructing proximal ureteral stones is disputed. We compare effectiveness and safety of flexible ureteroscopic lithotripsy (FURL) and tubeless percutaneous nephrolithotomy (TPNL) in treatment of upper ureteral stones larger than 1.5 cm. Methods: We reviewed the medical records of patients who performed FURL or TPNL for upper ureteral stones between June 2016 and November 2018. Comparative analysis was conducted regarding demographic parameters, stone free rate, postoperative pain and complications. Results: This study included 58 patients treated with FURL and 60 patients treated with TPNL owing to upper ureteral stones larger than 1.5 cm. Stone size was similar in the FURL and TPNL groups (17.6 ± 2.6 vs. 18.0 ± 2.1 mm, *p* = 0.194). The overall 3-month stone clearance rate was 95.8% for FURL versus 96.0% for TPNL (*p* = 0.575). There was no difference between the FURL and TPNL groups for hospital stay (*p* = 0.280) and postoperative complications. On the other hand, patients treated with FURL had longer operative time (*p* = 0.012) and less postoperative pain (*p* = 0.008). Conclusions: Both surgical techniques were considered feasible and effective surgical procedures in the treatment of large upper ureteral stones.

## 1. Introduction

Urinary calculi, or urinary stones, are a frequently encountered medical condition in urology. They exhibit a prevalence of approximately 2–3% in the overall population. Both prevalence and incidence are on the rise, constituting a noteworthy health concern [1,2]. Currently, American Urological Association (AUA) guidelines recommend extracorporeal shock wave lithotripsy (ESWL), percutaneous nephrolithotomy (PNL), ureteroscopic lithotripsy (URSL), and flexible ureteroscopic lithotripsy (FURL) for treating non-obstructing proximal ureteral stones. Despite the wide use of ESWL, the method is limited by its lower stone free rate (SFR) and necessity to repeat procedures involving upper ureteral stones > 10 mm [3]. Similarly, PNL has been associated with increased rates of complications including bleeding, pain, and urinary leakage [4,5]. Thus, alternative minimally invasive modalities like FURL were adopted and quickly implemented.

Tubeless PNL (TPNL) is a modified technique using internal ureteral stents in place of nephrostomy catheters for urinary drainage. In contrast, totally tubeless PNL is a technique that does not use a nephrostomy catheter or a ureteral stent after performing PNL [6]. This technique reduced catheter-related morbidity, prolonged hospitalization, and postoperative pain and discomfort. FURL can minimize the risk factors associated with PNL, and post-op results with SFR were similar to those performed via PNL [7].

Due to the relative paucity of clinical information guiding the treatment of large non-obstructing proximal ureteral stones, they are particularly challenging to manage with minimally invasive techniques. Few studies exist comparing the efficiency and safety of TPNL and FURL in treating non-obstructing proximal ureteral stones > 15 mm.

The purpose of the present study was to assess and compare the efficacy and safety of FURL and TPNL for the treatment of non-obstructing proximal ureteral stones > 15 mm.

## 2. Materials and Methods

### 2.1. Patients

The present study has been approved by the Institutional Review Board of Saint Mary’s Hospital (Approval number: UC23RISI0135). The guidelines for ethics and data management corresponded to local standards and the study has been performed in accordance to the standards of the Declaration of Helsinki. In this retrospective data analysis, we reviewed the medical records of patients with non-obstructing proximal ureteral stones > 15 mm who were operated on between June 2016 and November 2018 in two Saint Mary’s Hospitals. All procedures were performed by two experienced endourologic surgeons (HJC, KSK). The procedures were chosen according to the preferences of the patients and the two endourologic surgeons. Proximal ureteral stones were defined as stones between the upper margin of the iliosacral joints and ureteropelvic junction. The inclusion criteria were having a non-obstructing proximal stone > 15 mm in length based on standard imaging, while patients with non-obstructing proximal ureteral stones in addition to other renal, ureteral, or bladder stones were excluded. The exclusion criteria were having contraindications for percutaneous renal surgery such as active urinary tract infections and coagulopathy. Preoperative patient evaluations included a history, physical examination, urine analysis, urine culture, complete blood count, serum biochemistry, coagulation test, ultrasonography, radiography of kidney, ureter, and bladder radiography (KUB), and abdomen-pelvic computed tomography (CT). If urine cultures were positive, all patients were adequately treated with appropriate antibiotics and had a negative urine culture before their operation. Stone size was determined by measuring length and width on preoperative KUB or CT. Anticoagulant medications were stopped three to seven days before surgery in patients previously treated with anticoagulant therapy. “Stone free” was defined as complete stone clearance or clinically insignificant residual stone fragments (<4 mm) observed on postoperative imaging 3 months later.

### 2.2. Surgical Procedure

The patients were properly placed in the lithotomy position for FURL under general anesthesia. First, a semirigid ureterscopy or cystoscopy was inserted to place a guidewire into the renal pelvis. A 11/13 Fr or 12/14 Fr ureteral access sheath (Uropass; Olympus Corp., Tokyo, Japan) was placed over the guidewire until it reached the level of the non-obstructing proximal ureteral stone, and the flexible video ureteroscope (UFR-V, Olympus Corp., Center Valley, PA, USA) was inserted through the ureteral access sheath. Lithotripsy was performed with a laser lithotripter (VersaPulse Powersuite 100 W, Lumenis, Tel Aviv, Israel). A nitinol stone basket (Zero Tip, Boston Scientific, Marlborough, MA, USA) was utilized to remove as many large, fragmented stones as possible. Dusted particles were not extracted to permit natural draining. Fluoroscopy was performed to assess stone clearance at the end of the surgery. A 6 Fr double J stent was inserted during the surgery and removed at 2 to 4 weeks postoperatively.

The surgical procedure of TPNL was described previously [8]. All TPNLs were performed under general anesthesia with the patients in the prone position. Tract dilatation was performed using a balloon dilator (X-Force Dilatation Balloon, Bard, New York, NY, USA) up to 24Fr under fluoroscopy. The stones were fragmented and extracted through 22Fr nephroscopy (Richard Wolf, Knittlingen, Germany). At the end of the procedure, anterograde pyelography was performed to confirm the clearance of stone fragments. If there were no intraoperative events or residual stone fragments, all instruments were removed, and a nephrostomy tube was not placed. The incision of the nephrostomy tract was sutured using 3-0 nylon, and manual compression was performed for 5 min. A double J stent was placed after completing the surgery in cases with the presence of an edematous or inflamed ureteropelvic junction, suspicious residual stone fragments, and/or mild bleeding.

### 2.3. Outcome Evaluation

The primary outcome was SFR, which was defined as the absence of stone fragments or only having asymptomatic fragments < 4 mm on KUB or CT at the 3-month follow up visit. Secondary outcomes included operative time, duration of hospital stay, and postoperative complications. Postoperative complications were classified according to modified Clavien–Dindo system [9].

### 2.4. Statistical Analyses

Statistical comparison of continuous variables were presented as mean ± standard deviation compared using either Student’s or Mann–Whitney U test. Categorical variables were expressed as percentages and compared using Chi-squared and Fisher’s exact test. *p* value < 0.05 was considered statistically significant. All statistical data analyses were conducted using SPSS version 19.0 (IBM Inc., Chicago, IL, USA)

## 3. Results

A total of 118 patients were included in this study, of whom 58 patients recieved FURL and 60 patients recieved TPNL. Patient demographics and stone characteristics of both groups are presented in Table 1. No significant difference was shown between both groups in age, BMI, gender ratio, and laterality. The average stone size of both groups was similar (17.6 ± 2.6 vs. 18.0 ± 2.1 mm, *p* = 0.060). Stone density and presence or absence of hydronephrosis were also similar between the groups. There was no statistically significant difference between the groups in the time from diagnosis to operation.

The mean operation time was significantly lower in the TPNL group than in the FURL group (*p* = 0.012). Postoperative hospital stay was 3.6 ± 0.9 vs. 3.2 ± 1.3 days (*p* = 0.280). The initial and three-month SFR were not statistically different between the two groups (89.7% vs. 90.0%, *p* = 0.415 and *p* = 0.574, respectively). The visual analog scale (VAS) scores obtained 8 h after operation were 3.4 ± 1.3 in the FURL group vs. 5.1 ± 1.5 in the TPNL group (*p* = 0.008). The average post-op day 1 mean VAS was 2.3 ± 1.1 in the FURL group vs. 3.5 ± 1.2 in the TPNL group (*p* = 0.280). No patients became symptomatic or received further treatment for residual stone fragments (Table 2).

The complications were classified according to the modified Clavien–Dindo system and are represented in Table 3. Complication rate (grade I and II) in the FURL group was 6.9%, including one case of mucosal injury, two cases of bleeding not requiring blood transfusion, and one case of urinary tract infection controlled by antibiotics. In the TPNL group, complication (grade I and II) rate was 6.7%, which was one case of mucosal injury and three cases of mild bleeding not requiring blood transfusion. No major complications such as septic shock or mortalities arised in either treatment group.

## 4. Discussion

Upper ureteral stones have become one of the most pervasive stone diseases in the modern era and pose a significant healthcare burden on our population. Long-term sequelua of upper ureteral stones include but are not limited to interruptions in urine flow that build back-pressure in the ureter and kidney, eventually resulting in hydronephrosis. The management of upper ureteral stones depends on the size of the stone, associated duration and intensity of pain, non-obstructed vs. obstructed urine drainage, as well as cost and availability of instruments [10]. Most upper ureteral stones require medical intervention due to their intrinsically low spontaneous expulsive rates [11]. Although small-caliber semi-rigid ureteroscope combined holmium laser lithotripsy is considered first-line for large proximal ureteral stones [3], large calculi in the proximal ureter may be embedded in significantly tortous ureters that are difficult to access using semi-rigid ureteroscopes.

Advancements in technique and technology in treating proximal ureteral stones have promoted impressively high SFRs and low morbidity rates. Bellman et al. [12] initially reported TPNL in 1997 where he demonstrated decreased analgesic requirements, hospital stay, and recovery to normal activity compared to conventional PNL. In 2004, Aghamir et al. [6] was the first to report a successful TPNL that did not use an indwelling double J stent. They compared 43 patients that either underwent TPNL or standard PNL with placement of a ureteral stent and a nephrostomy catheter. Based on 43 patients that either underwent TPNL or standard PNL with ureteral stent and nephrostomy catheter placement, the group concluded that TPNL offers shorter hospital stays, analegisc requirements, and faster return to activities of daily living. These results were later supported by several more studies that reinforced the effectiveness and safety of TPNL [13,14].

Since the first introduction of flexible ureteroscopy by Marshall in 1964 [15], flexible ureteroscopy, with its low morbidity and its use of a natural ureteral orifice, has been considered the gold standard as a minimally invasive modality in cases where PNL or ESWL are not feasible for upper urinary stones > 20 mm [16]. FURL provides a higher stone clearance rate with lower morbidity, a shorter hospital stay, less patient discomfort, and an earlier return to daily activities in cases involving larger stones [17]. There is no clear consensus for the treatment of impacted upper ureteral stones. ESWL, URL, PNL and laparoscopic surgery are current treatment options with different success rates and morbidities. Especially for impacted stones, the SFR of ESWL and URL are low, and if the stone size is ˃1 cm, the SFR decreases significantly [18]. Laparoscopic ureterolithotomy is recommended in the EAU guidelines for multiple or impacted ˃1.5 cm ureteral stones in which URL and ESWL have failed or are likely to fail. Single-center 10-year experience of laparoscopic ureterolithotomy for >15 mm impacted ureteral stones was reported [19]. The authors commented that laparoscopic ureterolithotomy can be regarded as the first surgical procedure for patients with impacted and large ureteral stones due to high success and low complication rates. Furthermore, it can also be considered a salvage option after failed ESWL and URL.

Guler et al. [20] performed a comparison study of retrograde intrarenal surgery and laparoscopic surgery for large (>1.5 cm) upper ureteral or renal pelvis stones. The authors concluded that laparoscopic surgery shows higher SFR and lower further treatment compare to retrograde intrarenal surgery. However, laparoscopic surgery can be not good in relation to surgical time, hospitalization stay, postoperative VAS scores, and analgenic use.

Several studies evaluated the clinical efficacy and safety of minimally invasive PNL (mPNL) and flexible ureteroscopy for the treatment of proximal ureteral stones. Hu et al. compared mPNL and FURL in 184 geriatric patients for the treatment of renal and/or proximal ureteral stones sized 10–20 mm. They concluded that mPNL is more efficacious for multiple ureteral stones, but FURL shows shorter postoperative hospital stay and fewer complications [21].

A prospective study to assess the difference between supine mPNL and FURL in the treatment of surgically indicated single, large proximal ureteral stones was performed in China, but the results demonstrated no significant difference in stone size between the groups [22]. The authors concluded that both mPNL and FURL are effective and safe surgical options for patitents with single large proximal ureteral stones. FURL is associated with faster recovery and less invasiveness than mPNL in the supine position. Furthermore, Jia et al. [23] reported the results of comparing super-mPNL and RIRS in managing pediatric patients with 1–2 cm upper ureteral stones and concluded that super-mPNL exhibits better SFR and lower re-treatment and complication rates compared to RIRS. Furthermore, comparison study for the treatment of large impacted proximal ureteral stones was executed between minimally invasive percutaneous ureterolithotripsy and retrograde ureterolithotripsy [24]. A total of 59 patients were included in the present study, of whom 30 were treated by mini-PNL and 29 were treated by URSL.The initial SFR revealed 93.3% in the mini-PNL group and 41.4% in the URL group (*p* < 0.001). However, the overall SFR at the 1-month followup after initial operation was 100% in the mini-PNL group and 89.7% in the URSL group (*p* = 0.07). Comparison study between retrograde URL and percutaneous anterograde URL for removal of impacted upper ureteral stone >1 cm in patients > 65 years of age was executed in 2021 [25]. The authors commented that the success rate of the anterograde URL was significantly higher than that of the retrograde URL group (*p* = 0.0007). The complication rates were similar for both groups (*p* = 0.86). They concluded that anterograde URL should be considered as one of the primary surgical modalities for treatment of impacted upper ureteral stones in elderly patients.

A meta-analysis reviewed the literature for large upper ureteric stones  > 1 cm managed by various treatment options (laparoscopic ureterolithotomy, PNL, URL, and ESWL) and showed that PNL was superior to URL and ESWL for SFR and the need for auxillary procedures. Furthermore, duration of hospital stay was significantly shorter for URL than PNL from network analysis, but there was no significant difference for the rest of the comparisons. The authors concluded that this study was plagued by the poor quality of included studies. Thus the choice of a treatment modality in a given situation will continue to depend upon patient- and surgeon-driven factors until a good-quality randomized controlled study comparing all the four surgical procedures for large upper ureteric stones comes up [26]. Another meta-analysis study was performed to investigate the safety and efficacy of minimally invasive surgical ureterolithotomy in comparision with URL for the treatment of large ureteric stones. The SFR at discarge and 3-month follow up was higher with minimally invasive surgical ureterolithotomy with odds ratios of 6.30 and 5.34, respectively. Favorable results in terms of surgical time and hospital stay were obtained in the case of URL. The authors commented that both URL and minimally invasive surgical ureterolithotomy could be considered for the treatment [27].

While the studies discussed above have provided the field with invaluable information, literature comparing FURL and TPNL is sparse. To address this research gap, we conducted this study by comparing TPNL to FURL for upper ureteral stones larger >15 mm. The TPNL group had a shorter operation time (65.6 vs. 41.5 min, *p* = 0.012) than the FURL group. Duration of postoperative hospital stay was similar between the two groups (*p* = 0.280). In analyzing the data, we took into consideration that having a nephrostomy catheter for drainage posed as a confounding variable that adds time to the duration of hospital stay due to the need for post-op in-patient recovery. Accordingly, we adjusted for this variable and concluded that the duration of hospital stay in the TPNL group was not significantly different than in the FURL group.

The initial SFR was 78% in the FURL group and 91% in the TPNL group (*p* < 0.05). The overall SFR at the 3-month follow-up visit after initial treatment was 95.8% in the FURL group and 96.7% in the TPNL group (*p* = 0.574). A literature review of studies on the treatment of proximal ureteral stones is presented in Table 4.

The results of our study suggest that FURL with modern techniques and technology can accomplish the same satisfactory results as TPNL. While TPNL may not be suitable for select patients who are being treated with anticogualant therapy for bleeding disorders, FURL provides the added benefit of being both safe and effective in said patients [33].

Legemate et al. [34] reported the SFR and complications of URL for impacted ureteral stones compared with non-impacted ureteral stones. A total of 2650 (31.0%) patients were treated for impacted stones and 5893 (69.0%) for non-impacted stones. The overall SFR was 87.1% for impacted ureteral stones, which is significantly lower compared with 92.7% for non-impacted stones. Significantly higher ureteral perforation and avulsion rates were showed in the impacted stone group compared with the non-impacted stone group. The authors concluded that URL for impacted stones showed lower SFR and higher intra-operative complication rates compared with URL for non-impacted stones. Interestingly, our study did not contain impacted proximal ureteral stones. We do not know the exact reason why there were no impacted stones during this present study; it may have been because there were not many surgical cases.

Our study has several limitations. The first limitation was the study’s retrospective design and lack of randomization. A large number of prospective randomized controlled studies should still be conducted in the future to confirm the effectiveness and safety of both surgical techniques. Our sample size was relatively small, and the follow-up duration was also short. Thus, we may not have encountered long-term sequalae like ureteral stenosis. Despite these shortcomings, this study provides invaluable data that can begin to address the research gap regarding the comparison of TPNL and FURL. Second, the duration of hospital stay for both groups in this study was longer than that of similar studies. This could be influenced not only by the surgical results but also by our patients’ preferences. Patients in Korea tend to extend the duration of their hospital stays due to low institutional costs implemented by National Health Insurance in Korea.

## 5. Conclusions

Both TPNL and FURL are relatively safe and effective in the treatment of proximal ureteral stones size > 15 mm. Although FURL showed longer operative time compared with TPNL, both surgical techniques have comparable higher SFR and complications. On the other hand FURL has the advantage of less postoperative pain. In conclusion, our study demonstrates a great potential for the implementation of TPNL and FURL in current clinical practice.

## Figures and Tables

**Table 1:** Characteristics of patients.

	FURL	TPNL	*p* Value
**No of procedure**	58	60	
**Age (years)**	52 ± 9.5	55.9 ± 13.7	0.222
**BMI (kg/m^2^)**	20.5 ± 3.1	21.1 ± 2.5	0.554
**Gender**			0.143
Male	35	39	
Female	13	11	
**Laterality**			0.397
Right	23	29	
Left	25	21	
**ASA grade**			0.417
1	13 (27.1%)	15 (30.0%)	
2	23 (47.9%)	25 (50.0%)	
3	12 (25.0%)	10 (20.0%)	
**Stone diameter (mm)**	17.6 ± 2.6	18.0 ± 2.1	0.194
**Stone density (HU)**	1124 ± 120.7	1194 ± 223.4	0.304
**Hydronephrosis**	45 (93.8%)	48 (96%)	0.618

FURL, flexible ureteroscopic lithotripsy; TPNL, tubeless percutaneous nephrolithotomy; Notes: continuous variables are expressed as mean and standard deviation in parentheses. Categorical variables are expressed as counts and percentages in parentheses.

**Table 2:** Comparison of perioperative data and outcomes.

	FURL	TPNL	*p* Value
**Operative time (min)**	65.6 ± 46.4	41.5 ± 22.0	0.012
**Postoperative hospital stay (d)**	3.6 ± 0.9	3.2 ± 1.3	0.280
**Change in Hb level (g/mL)**	1.1 ± 0.4	1.5 ± 0.1	0.407
**Transfusion**	0	0	NA
**Initial SFR**	52 (89.7%)	54 (90.0%)	0.415
**3-month SFR**	56 (95.8%)	58 (96.7%)	0.574
**VAS score**			
Day 0	3.4 ± 1.3	5.1 ± 1.5	0.008
Day 1	2.3 ± 1.1	3.5 ± 1.2	0.280

FURL, flexible ureteroscopic lithotripsy; TPNL, tubeless percutaneous nephrolithotomy; Hb, hemoglobin; SFR, stone free rate; VAS, visual analogue scale.

**Table 3:** Complications according to the Clavien grading system.

	FURL	TPNL	*p* Value
**Grade I**			
Mucosal injury	1	1	0.610
Bleeding not required transfusion	2	3	0.109
**Grade II**			
Bleeding required transfusion	0	0	NA
UTI managed by antibiotics	1	0	0.156
**Grade IIIa**			
Urinary leakage	0	0	NA
Ureteral perforation	0	0	NA
**Grade IIIb**			
Bleeding managed by angioembolization	0	0	NA
**Grade IV**			
Sepsis	0	0	NA

FURL, flexible ureteroscopic lithotripsy; TPNL, tubeless percutaneous nephrolithotomy.

**Table 4:** Literature review of studies on treatment proximal ureteral stones.

Study	Procedure	Patient Number	Stone Size (mm)	SFR (%)	Operative Time (min)	Hospital Stay (day)	Complications
Wang et al. [28]	Mini-PNL	50	19.4	96	125	6.8	3 transfusions
	URL	50	16.8	72	55	2.5	2 strictures
Liu et al. [29]	PNL	45	14.6	97.8	53.8	6.8	4.4% fever, 2.2% stricture
	URL	45	14.8	82.2	60.1	5.2	4.4% fever, 4.4% stricture, 2.2% perforation
Basiri et al. [30]	PNL	50	20.3	86	42.7	2.8	6% urine leakage
	URL	50	17.8	76	93.6	2.9	None
Mousavi et al. [31]	PNL	52	18	100	32	2.1	25% fever, 3.8 transfusion
	LUL	55	21	100	107	2.1	9.1% fever, 3.6% urine leakage
Choi et al. [32]	URL	52	22	73.1	49.7	4.9	1 urine leakage
							1 stricture
	LUL	48	21	100	128.5	6.7	2 urine leakage
							1 stricture

SFR, stone free rate; URL, ureteroscopic lithotripsy; PNL, percutaneous nephrolithotomy; LUL, laparoscopic ureterolithotomy.

## Data Availability

The data presented in this study are available on request from the corresponding author.
